# Percutaneous Treatment Approaches in Atrial Fibrillation: Current Landscape and Future Perspectives

**DOI:** 10.3390/biomedicines10092268

**Published:** 2022-09-13

**Authors:** Panagiotis Theofilis, Evangelos Oikonomou, Alexios S. Antonopoulos, Gerasimos Siasos, Konstantinos Tsioufis, Dimitris Tousoulis

**Affiliations:** 1 First Department of Cardiology, “Hippokration” General Hospital, University of Athens Medical School, 11527 Athens, Greece; 2 Third Department of Cardiology, Thoracic Diseases General Hospital “Sotiria”, University of Athens Medical School, 11527 Athens, Greece

**Keywords:** atrial fibrillation, catheter ablation, left atrial appendage closure

## Abstract

Atrial fibrillation (AF), the most common sustained arrhythmia in clinical practice, represents a major cause of morbidity and mortality, with an increasing prevalence. Pharmacologic treatment remains the cornerstone of its management through rhythm and rate control, as well as the prevention of thromboembolism with the use of oral anticoagulants. Recent progress in percutaneous interventional approaches have provided additional options in the therapeutic arsenal, however. The use of the different catheter ablation techniques can now lead to long arrhythmia-free intervals and significantly lower AF burden, thus reducing the rate of its complications. Particularly encouraging evidence is now available for patients with persistent AF or concomitant heart failure, situations in which catheter ablation could even be a first-line option. In the field of stroke prevention, targeting the left atrial appendage with percutaneous device implantation may reduce the risk of thromboembolism to lower rates than that predicted with conventional ischemic risk scores. Left atrial appendage occlusion through the approved Watchman or Amplatzer devices is a well-established, efficacious, and safe method, especially in high-ischemic and bleeding risk patients with contraindications for oral anticoagulation.

## 1. Introduction

The prevalence and incidence rates of atrial fibrillation (AF), the most common sustained arrhythmia in clinical practice, are constantly rising, especially in developed, high-income countries [1]. Over 400 new cases per million persons were reported in 2017, displaying a significant increase compared to the 345 and 309 new cases per million persons that were observed in 2007 and 1997, respectively [1]. As a result, the worldwide AF prevalence is approximately 37.574 million cases, representing a 33% increase compared to 1997 [1]. Additionally, there has been a 105% increase in deaths attributable to AF from 1997 to 2017 (0.51% of overall mortality) [1]. These trends will continue to predominate in the upcoming decades, with an absolute increase in incidence and prevalence of approximately 63% and 66%, respectively [1]. Major modifiable and non-modifiable risk factors should be taken into account for this increase, such as population ageing, female sex, arterial hypertension, obesity, diabetes mellitus, and genetics, among others [2,3,4,5].

AF is responsible for important life-threatening complications, such as embolic stroke and heart failure (HF) [6,7], and studies have also shown a decrease in cognitive function in subjects with AF [8]. Ultimately, individuals with AF may have depression and impaired quality of life [9,10], as well as a higher rate of hospitalizations and mortality [11]. However, the institution of novel therapeutic approaches is believed to ameliorate patient prognosis. In particular, interventional approaches are gaining ground in the management algorithms, owing to the encouraging data from clinical trials. This review aims to summarize the current knowledge and applications of contemporary percutaneous procedures in the management of AF, namely catheter ablation (CA) and left atrial appendage closure (LAAC).

## 2. Atrial Fibrillation Catheter Ablation

Early rhythm control is essential in reducing the burden of AF complications [12], regardless of the presence of symptoms [13]. Percutaneous catheter AF ablation constitutes an attractive approach towards rhythm control. Radiofrequency (RF) CA, in particular, is the most commonly performed ablation procedure in electrophysiology. It predominantly consists of pulmonary vein isolation (PVI), as these are considered major initiators of paroxysmal AF [14]. After this first description in 1998, segmental ostial PV ablation was introduced and was bolstered by the use of 3D electroanatomical mapping [15]. Later on, wide-area circumferential ablation with verification of the conduction block was proven superior than ostial segmental isolation of each individual pulmonary vein [16,17], through the neutralization of more proximal trigger sources and providing concomitant autonomic denervation [18]. Consequently, antral ablation is the main AF ablation technique and is recommended during all AF ablation procedures [19]. In CA procedures for AF, uninterrupted periprocedural anticoagulation with direct oral anticoagulants is suggested [20]. Moreover, the use of intracardiac echocardiography could also be of use for reducing fluoroscopy time, procedure duration, and the rate of complications [21].

Regarding clinical outcomes, CA reduces the risk of stroke/transient ischemic attack (risk ratio (RR) 0.61, 95% CI 0.39–0.97, *p* = 0.035) and death (RR 0.7, 95% CI 0.55–0.89, *p* = 0.004), compared to medical treatment [22]. Moreover, it may lead to a greater rate of sinus rhythm maintenance (RR 3.55, 95% CI 2.34–5.40, *p* < 0.001) and improve the left ventricular ejection fraction (weighted mean difference (WMD) 5.39, 95% CI 2.45–8.32, *p* < 0.001) [22]. Critically, CA for AF appears to be a cost-effective procedure, according to an analysis performed in the United Kingdom [23]. It should be noted that the large-scale CABANA randomized clinical trial, comparing CA to anti-arrhythmic drugs (AADs) in AF, was neutral regarding the primary composite end point of death, disabling stroke, serious bleeding, or cardiac arrest. Concerns about the lower-than-expected event rates and treatment crossovers have been raised, and, therefore, the results should be cautiously considered [24]. Moreover, age variations were detected, with younger age populations (<65 years of age) exhibiting the greatest relative and absolute benefits from the CA procedure [25].

In the landmark trial mentioned above, AF recurrence was still significantly reduced by CA compared to AADs during the 5-year follow-up, irrespective of AF type [26]. However, AF recurrence after CA is not uncommon. Several factors are believed to be implicated, including structural heart disease, left atrium characteristics (diameter, voltage), PV isolation-reconnections, AF duration, the presence of non-PV triggers, cardiometabolic risk factors, chronic kidney disease, and anxiety [27,28,29]. Interestingly, AF ablation in the CABANA randomized clinical trial led to reductions in left atrial volume index and mean PV ostial diameter, which were associated with lower AF recurrence rates [30]. Biomarkers have also been associated with AF recurrence after CA, namely N-Terminal-pro-B-type-natriuretic peptide, B-type natriuretic peptide, high-sensitivity C-reactive protein, carboxy-terminal telopeptide of collagen type I, interleukin-6, and galectin-3 [31,32]. However, their exact role in patients undergoing CA for AF needs to be precisely determined.

As endorsed by the latest guidelines from the European Society of Cardiology and the European Heart Rhythm Association, published in 2020, CA is now a mainstay of AF rhythm control strategies [33]. It is recommended as a first line treatment option in patients with suspected AF-induced cardiomyopathy. It should be considered in individuals with symptomatic paroxysmal AF or with concomitant AF and HF with a reduced left ventricular ejection fraction. Lastly, it can be considered in patients with persistent AF in the absence of risk factors for recurrence. Moreover, in cases of drug failure or intolerance, CA is recommended irrespective of the AF type.

### 2.1. Catheter Ablation Types

#### 2.1.1. Radiofrequency Ablation

The effectiveness of RF ablation has been established. This procedure was associated with greater arrhythmia-free survival (RR 0.62, 95% CI 0.51–0.74, *p* < 0.001) and less hospitalizations (RR 0.32, 95% CI 0.19–0.53, *p* < 0.001) compared to AADs in a recent meta-analysis [34]. Other than the effectiveness in reducing major endpoints, a reduced risk of incident dementia with AF CA was proposed by Saglietto et al. in their systematic review and meta-analysis [35]. Moreover, it may also prevent the progression of paroxysmal to persistent AF [36]. This is particularly important, since such patients may be facing an adverse cardiovascular prognosis, as previously shown [37]. It should be noted that same-day discharge may be feasible, safe, and effective in selected patients [38]. Ablation guidance through unipolar signal modification may improve PVI outcomes in terms of lower radiofrequency time and AF recurrence at 12 months, as shown in a randomized clinical trial of 136 patients with paroxysmal AF [39].

The electrical reconnection of at least one PV may influence the arrhythmia-free interval after an RF ablation procedure. Interestingly, it should be noted that the presence of electrical reconnections in those patients undergoing a redo RF ablation may be a good predictive sign compared to those without PV reconnections, as the number of reconnected PVs was independently related to lower AF recurrence in a mean 18.4-month follow-up (hazard ratio (HR) 0.56, 95% confidence interval (CI) 0.34–0.95, *p* = 0.032) [40]. To manage PV reconnection, operators could opt for a contact force of 5–10 g, and assess for the loss of capture over the ablation line, confirm the exit block, and perform adenosine testing [19]. Improvement in mapping techniques may further support the successful PVI with the use of high-density multielectrode catheters, providing more precise electroanatomical maps that reflect real-time volume-rendered left atrium and PV geometry during ablation. In a recent study, the high-density grid catheter, consisting of four splines, each with four small (1 mm), equally spaced (3 mm) electrodes, was able to detect a significantly higher number of PV reconnections compared to the standard bipolar mapping [41]. For the lesion size, minimum ablation indexes (AI) of ≥370 and ≥480 for posterior-inferior and anterior-superior segments, respectively, should be sought [42]. Other protocols, such as the CLOSE protocol which incorporates AI, inter-lesion distance, and catheter stability, have been proven reliable in achieving adequate PVI with an impressive rate of freedom from atria tachyarrhythmia [43]. Moreover, lesion size index is another metric attributed to the TactiCath ablation catheter, with higher values being associated with a shorter procedure, radiofrequency, and fluoroscopy times, as well as fewer touch-up ablations compared to the low lesion size index [44].

**Figure 1:**
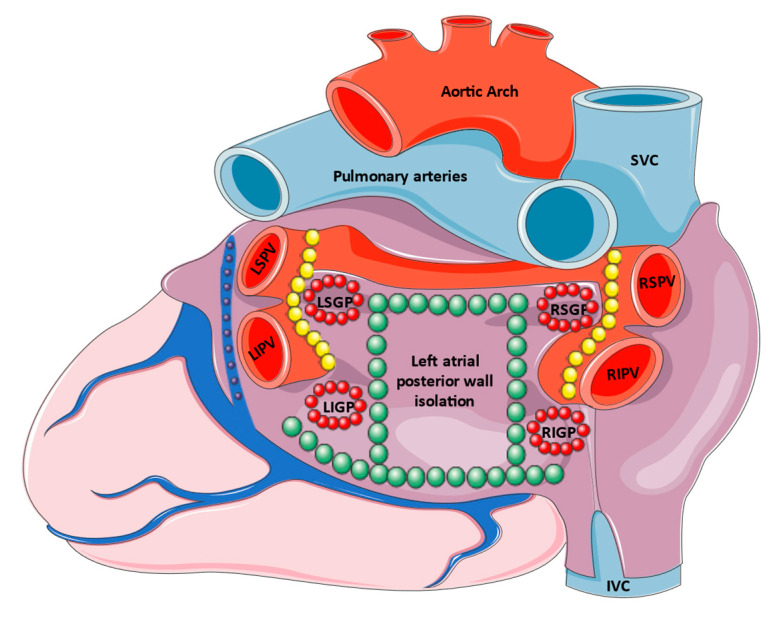
Radiofrequency catheter ablation of AF triggers. Although pulmonary vein isolation (yellow circles) remains the cornerstone of AF ablation, the ablation of the left atrial posterior wall (green circles), ganglionated plexuses (red circles), and retrograde ethanol infusion in the vein of Marshall (purple circles) are additional options that may be considered. IVC: inferior vena cava, LIPV: left inferior pulmonary vein, LIGP: left inferior ganglionated plexus, LSPV: left superior pulmonary vein, LSGP: left superior ganglionated plexus, RIGP: right inferior ganglionated plexus, RSGP: right superior ganglionated plexus, RIPV: right inferior pulmonary vein, RSPV: right superior pulmonary vein, SVC: superior vena cava.

Regarding the additional ablation of AF triggers (Figure 1), the performance of circumferential PVI together with an electrical posterior box isolation in subjects with reconnected PV did not confer any additional efficacy regarding arrhythmia-free survival [45]. Posterior left atrial wall isolation is under investigation in other ongoing clinical trials of AF ablation [46,47], with the results being expected to provide essential information. Regarding other ablation sites, targeting ectopy-triggering ganglionated plexuses was equally effective to PVI in patients with paroxysmal AF, with a trend towards a lower rate of atrial arrhythmia prevention and significantly lower AAD usage after the procedure [48]. This finding was further supported by a meta-analysis, which showed a lower arrhythmia recurrence (odds ratio (OR) 0.58, 95% CI 0.41–0.82) that was dependent on the left atrial diameter in a meta-regression analysis [49]. Lately, Li et al. showed that the vein of Marshall ethanol infusion decreased atrial tachyarrhythmia recurrence compared to PVI alone, especially when substrate ablation was also performed [50].

A modified high-power, short-duration (HPSD) RF ablation protocol has also been proposed, aiming to improve lesion-to-lesion uniformity and produce a transmural injury [51]. In the earliest study of Nilsson et al., the use of power output at 45 W for 20 s resulted in a greater need for more RF applications to achieve adequate PVI, with a shorter procedural time compared to a 30 W/120 s duration conventional CA [52]. In a subsequent randomized trial, HPSD was associated with a lower number of ablations needed for PVI, however [53]. HPSD ablation appears to be a more durable procedure, with lower rates of chronic PV reconnection, owing to enhanced catheter stability [54]. This finding was not accompanied with lower rates of arrhythmia-free intervals, however. This could be associated with progressive fibrous atrial cardiomyopathy, which may be evident in the long term after HPSD [55]. Posterior box and superior vena cava isolation are also feasible with a HPSD ablation method [56,57]. Additionally, HPSD is an appropriate option in redo CA procedures, as shown by Junarta et al. [58]. According to meta-analytic evidence, HPSD ablation results in a greater rate of freedom from atrial arrhythmia (OR 1.44), decreased acute PV reconnection (OR 0.56), and ameliorated first-pass PVI (OR 3.58) [59]. Moreover, the procedural, fluoroscopy, and ablation times were all significantly reduced, paired with similar rates of complications compared to conventional RF ablation [59]. Concerning the procedural safety, the operators should avoid the delivery of lesions in quick succession, as this may induce esophageal overheating and injury. Esophageal thermal injury is more common in subjects undergoing HPSD RF ablation procedures, but are not related to esophageal lesions in esophagogastroduodenoscopy [60,61,62]. Even in subjects exceeding luminaλ esophageal temperatures of 39 °C, there were no esophageal perforations or atrial–esophageal fistulas after upper endoscopic evaluation [63]. AI guidance or the CLOSE protocol could be safely used in HPSD ablation procedures, as previously shown [64].

The very high-power, short-duration (vHPSD) RF ablation technique involves a power output of 90 W for 4 s, with acceptable safety and efficacy proven in the QDOT-FAST trial [65], subsequently confirmed in the fast and furious AF study [66]. In a study with slightly lower thresholds (70 W/7 s), vHPSD led to a greater rate of arrhythmia-free intervals at 1 year, as well as decreased RF and procedural time, compared to the conventional procedure [67]. When incorporating temperature control during vHPSD ablation, there was no evidence of postprocedural esophageal ulceration, with silent cerebral events being noted in 24% of patients [68].

#### 2.1.2. Cryoablation

Other than RF ablation, cryoablation through the use of a cryoballoon is a valid alternative approach. This method aims to create intramural, irreversible myocardial lesions, and is characterized by a short learning curve [69]. Regarding this technique, the single application of the cryoballoon at −40 °C and the achievement of isolation within 60 s are predictors of adequate electrical PVI [70]. Moving to clinical outcomes, in a recently reported randomized clinical trial of patients with symptomatic, paroxysmal AF, cryoablation performance was associated with a significantly lower atrial tachyarrhythmia recurrence (HR 0.48, 95% CI 0.35–0.66, *p* < 0.001) [71], confirming other lines of evidence [72,73]. Moreover, this technique may also lead to significant improvement in the quality of life, as well as to symptom resolution [74,75]. However, early arrhythmia recurrence in the 3-month blanking period was associated with a lower arrhythmia-free survival in patients undergoing cryoballoon ablation [76]. Comparative studies between RF and cryoablation have been performed to assess the most effective and safe procedure. In a recently reported randomized clinical trial of 346 patients with paroxysmal AF, there was no difference in the incidence of symptomatic atrial arrhythmia with the use of contact force–guided RF ablation, 4-min cryoablation, or 2-min cryoablation [77]. Moreover, all of those regimens appeared equally safe, with RF ablation being a longer procedure with shorter fluoroscopy time [77]. Interestingly, cryoballoon ablation also had a shorter procedural duration compared to HPSD RF ablation [78]. A recent analysis also pointed to potential sex-related differences in the outcomes between cryoballoon and RF ablation, with men exhibiting a better efficacy with cryoablation, with procedure failure defined as the recurrence of atrial arrhythmia, reablation, and reinitiation of anti-arrhythmic medication [79].

#### 2.1.3. Laser Balloon Ablation

Laser balloon (LB), through the use of point-by-point laser energy and covering 30° of a circle with each lesion, is another alternative with equal efficacy and safety to RF and cryoballoon ablation techniques [80,81,82]. Interestingly, visually-guided LB ablation was associated with lower atrial arrhythmia recurrence at 12 months compared to RF ablation in one study, with a negative adenosine provocation test being predictive of a positive outcome [83]. This could be related to a more complete PVI isolation and a lesser extent of fibrosis, as demonstrated by a previous cardiac magnetic resonance imaging study [84]. Additionally, a wide-area circumferential LB ablation led to a greater arrhythmia-free interval compared to a wide-area circumferential RF ablation in a retrospective multicenter study [85]. Moreover, a manual dragging laser irradiation technique could shorten the procedure duration without compromising efficacy or safety, as shown in the study of Sasaki et al. [86]. Third-generation LB ablation is characterized by a shorter laser application and procedure duration compared to second-generation, with a similar effect [87]. In another study of patients requiring redo procedures with second- and third-generation LB due to AF recurrence, persistent PVI was more frequent in the third-generation LB [88].

#### 2.1.4. Pulsed Field Ablation

The application of electroporation in the context of pulsed field ablation (PFA) is an evolving method in the field of PVI through CA. By inducing irreversible electroporation, durable lesions may be formed due to the loss of cellular homeostasis. Moreover, since cardiomyocytes have low sensitivity to PFA, a minimal risk of collateral damage is present. As shown in a recent imaging study, PFA results in an increased late gadolinium enhancement without injuring the microvasculature or inducing intramural hemorrhage, whereas the chronic fibrotic changes are of a lesser magnitude compared to thermal ablation techniques [89]. Additionally, the lack of coagulative necrosis with PFA suggests a minimal risk of incident pulmonary vein stenosis, as demonstrated by Kuroki et al. [90]. Furthermore, a short procedural duration should be mentioned. PFA is also effective in the case of irregular surfaces, where electrode–tissue contact is not ideal, providing more uniform lesions compared to RF ablation [91].

PFA has been evaluated preclinically, with a good efficacy and safety profile [92]. Therefore, clinical data are now accumulating regarding its use in humans. Among the first published reports, Reddy et al. utilized the endocardial catheter, Farawave (Farapulse Inc., Menlo Park, CA, USA). Being the only validated PFA system, this 12F catheter has a distal portion consisting of five splines, each with four electrodes per spline, able to provide voltages between 900 and 2500 V. The PFA and fluoroscopy duration was 19 and 12 min on average, respectively. The PVI was successful in all 15 patients included in this study [93]. According to the IMPULSE and PEFCAT trials that followed, there was a durable PVI in 100% of the procedures at the 3-month follow-up, whereas the freedom from AF was estimated at 87.4% at 1 year [94]. Moreover, the rate of short- and mid-term adverse events was very low. In the latest report of the MANIFEST-PF registry, PVI with PFA was accomplished in 99.9% of patients, with a low rate of major complications [95]. However, antral PVI may not be adequately achieved through PFA, since an insufficient isolation of the left anterior antral PV segments and enlarged LA isolation areas on the posterior segments was detected by Bohnen et al. [96]. Head-to-head trials comparing the different AF CA techniques are essential to demonstrate differences in the efficacy and safety, and to decide on the optimal ablation method.

Other PFA catheters are also available at earlier stages of development, however. To begin with, the circular PVI catheter by the name of PulseSelect (Medtronic, Minneapolis, MN, USA) possesses nine electrodes for bipolar, biphasic energy delivery and electrogram recording, while also being connected to a custom-built PFA or to an RF generator. In the recently published PULSED-AF pilot trial, electrical isolation was achieved in all 152 PV, with a mean procedure duration of 160 min, and without adverse events [97]. Moving to the lattice-tip ablation catheter with a compressible 9-mm nitinol tip, its ability to provide either PFA or RF ablation depending on the morphology of the desired lesion has been demonstrated in a recent pilot study [98]. Finally, a novel multipolar PFA catheter with the ability of real-time PV signal recording appears promising, with similar accuracy to the standard Farawave 3D-mapping system in detecting PVI and residual PV conduction [99]. However, large-scale studies are needed to obtain high-quality evidence for the importance of those systems.

#### 2.1.5. Hot Balloon Ablation

CA of AF through the use of an RF hot balloon is another alternative approach to RF ablation, with early studies showing appropriate PVI, as well as posterior left atrium isolation. A 92% freedom from arrhythmia without the need for AADs, after a mean 11-month follow-up period, was reported [100]. In the long-term, this technique is associated with freedom from atrial tachyarrhythmias in 64.7% of the cases after a 6.2-year follow-up [101]. Re-ablation was mostly required for the PV and posterior left atrium, with the rate of freedom from atrial tachyarrhythmias being 84.5% during the 4.6-year follow-up. PV stenosis and phrenic nerve palsy were infrequent. A single-shot energy application protocol for PV antrum ablation, sparing the PV ostium, has also been tried, with a high rate of sinus rhythm maintenance at 1 year [102]. Some additional hints may enhance the efficacy and safety of the procedure. Real-time balloon surface temperature monitoring when performing single-shot PVI is useful, as temperatures above 58.7 °C may be indicative of appropriate PVI [103]. Regarding safety, esophageal cooling with an infusion of lopamidol and saline in cases of luminal esophageal temperature exceeding 39 °C should be considered to reduce the risk of esophageal injury [104]. Lastly, we should note that although the efficacy of the hot balloon and cryoballoon in terms of AF recurrence is similar, a greater need for touch-up ablation was noted with the hot balloon, especially in left superior pulmonary veins [105]. This was accompanied by a significantly longer procedure duration. A balloon temperature of 73 °C in left superior pulmonary vein ablation may improve the outcome and eliminate the need for touch-up ablation in this region [106].

### 2.2. Catheter Ablation in Persistent Atrial Fibrillation

Regarding patients with persistent AF, long-term follow-up (~54 months) revealed that RF ablation may reduce the risk of ischemic cerebrovascular events and congestive HF, together with improvements in quality of life, when compared to pharmacotherapy [107]. LB ablation is another alternative in patients with persistent AF, showing similar rates of atrial tachyarrhythmia recurrence to cryoablation in a recently reported propensity-matched analysis [82]. Additional ablation of mapped low voltage areas on top of circumferential PVI did not seem to provide an incremental benefit at 18 months in a randomized trial of patients with persistent AF, since no difference in arrhythmia-free interval was documented [108]. However, in another study, low-voltage area substrate modification in patients with persistent AF undergoing CA resulted in greater arrhythmia-free survival, without any difference in procedure duration or periprocedural complications [109]. Complex-fractionated atrial electrograms (CFAEs) are electrograms with highly fractionated potentials or with a very short cycle length (<120 ms), which represent potential AF substrate sites requiring ablation. The combination of CFAE ablation with high-density voltage mapping resulted in higher rates of freedom from AF compared to PVI alone [110]. Further documentation of CFAE ablation efficacy was present in another study, where the combination of PVI, CFAE, and linear ablation resulted in a greater freedom from atrial arrhythmia in comparison to sole PVI (HR 1.56, 95% CI 1.04–2.34) [111]. Other studies failed to show a difference in 1-year freedom from atrial tachyarrhythmia with CFAE ablation, however [112,113]. This could be attributed to the focal energy sources being located mostly at the border or even outside of the CFAE areas, with those unablated sites potentially leading to AF recurrence [114]. Active CFAEs should be, therefore, distinguished from bystanding CFAEs, potentially through the use of nonlinear recurrence quantification analysis [115]. Active CFAEs characteristically present with an increase in electrogram conformation recurrence [115]. Future adequately designed studies might improve our knowledge in this regard.

Moving to the ablation of posterior atrial wall box isolation on top of circumferential PVI, no effect on the clinical outcomes was noted [116]. However, a recent meta-analysis showed that posterior atrial wall isolation could reduce AF recurrence compared to the control group in patients with persistent AF [117]. Valderrábano et al. investigated the retrograde ethanol infusion in the vein of Marshall, which is perceived as an AF triggering point, and showed that patients with persistent AF randomized to this procedure had a lower rate of atrial tachyarrhythmia recurrence and AF burden compared to conventional RF ablation, without any excess adverse events [118]. Interestingly, thoracoscopic ablation was not proven superior in the group of patients with persistent AF compared to CA regarding freedom from atrial tachyarrhythmia, with the latter being more cost-effective, improving patient symptoms, and providing more quality-adjusted life-years [119]. Lately, magnetic resonance imaging-guided atrial fibrosis ablation, together with PVI, was attempted in patients with persistent AF [120]. The study, however, did not achieve a greater arrhythmia-free interval, and the evaluated procedure was accompanied with more adverse events [120].

Focal, organized rotational activity believed to sustain AF and targeted ablation procedures have, thus, been attempted. This technique may not be appropriate as a sole strategy for paroxysmal AF, since a focal impulse and rotor mapping (FIRM)-guided rotor ablation resulted in shorter arrhythmia-free interval compared to cryoballoon or RF PVI in patients with paroxysmal AF [121,122]. In a study of 58 patients with nonparoxysmal AF, a single FIRM-guided rotor ablation, all patients had identifiable stable atrial rotors [123]. Their ablation resulted in atrial tachyarrhythmia freedom in 73.1% of the patients at 1-year follow-up [123]. FIRM-guided ablation may also be superior to linear left atrial ablation and CFAE ablation regarding the recurrence of AF, as shown by Hsieh et al. [124]. However, a meta-analysis of head-to-head studies comparing PVI with and without FIRM-guided ablation showed no statistically significant difference in atrial tachyarrhythmia recurrence at a mean 18.8 months of follow-up [125]. As the importance of this method is not well established, further evidence from randomized clinical trials may aid us in determining the role of this procedure in persistent AF ablation, possibly through identifying subgroups that might specifically benefit from it.

Dominant frequency (DF) assessment could potentially be of importance in identifying AF-driving regions. However, in the RADAR-AF trial, high-DF area ablation did not have an additive efficacy compared to PVI alone in patients with persistent AF [126]. A recent study found that high-DF areas frequently overlap with low-voltage areas, which are associated with AF recurrence after PVI in patients with nonparoxysmal AF [127]. Ablation of these overlapping areas in conjunction with PVI may lead to greater arrhythmia freedom rates, whereas this decreased in the PVI-only group proportionally to the extent of the low-voltage areas [128]. Since DF is spatiotemporally unstable, the study of Li et al. highlighted that high-DF pattern recurrence is associated with a greater degree of organization [129]. Thus, ablating these recurring patterns may improve the outcomes in patients with persistent AF. Since the optimal use of high-DF patterns in the ablation strategies has not been determined, further studies are required.

In a recently reported Bayesian network meta-analysis of 3394 persistent AF patients and 22 ablation strategies, a strategy involving PVI with left atrial posterior wall and non-PV trigger ablation was characterized as the most effective regarding freedom from atrial tachyarrhythmia recurrence [130]. Concomitant hybrid surgical and CA could also emerge as the most effective alternative in patients with nonparoxysmal AF, despite the increased rate of major adverse events and procedure duration [131]. These observations were mostly based on the landmark CONVERGE randomized controlled trial, which evaluated 149 patients with persistent AF who underwent either a minimally invasive epicardial/endocardial ablation approach or CA. After 1 year of follow-up, the hybrid convergent technique led to significantly lower rates of atrial tachyarrhythmia recurrence, whereas 74% had a ≥90% AF burden reduction [132]. It is clear, however, that the achievement of a favorable outcome in the group of patients with persistent AF is challenging, and further adequately designed studies are required to improve our understanding regarding the optimal ablation strategy.

Finally, we should mention that the evolution of cardiac mapping with novel high-density mapping systems using multielectrode catheters could further improve the outcomes of AF CA in the future, especially in the setting of nonparoxysmal AF. Available systems, such as the Rhythmia HDx ultra-HD, the EnSite Precision, the CARTO 3, and the noncontact AcQMap High Resolution Imaging and Mapping System, may improve AF substrate delineation, greatly improve the efficiency of the mapping procedure without affecting safety, and provide additional AF mechanistic insights [133].

### 2.3. Catheter Ablation in Heart Failure

CA of AF in HF patients has gained popularity after the presentation of the landmark CASTLE-AF trial (Table 1). Patients with symptomatic AF, HF and a left ventricular ejection fraction of <35%, New York Heart Association class II-IV, and an implantable defibrillator were enrolled. A previous failed response to AADs, the presence of important side effects, or an unwillingness to take these agents were other inclusion criteria. Patients who underwent the CA procedure had a lower risk of facing the composite endpoint of all-cause mortality or HF hospitalization (HR 0.62, 95% CI 0.43–0.87, *p* = 0.007), after a median 37.8-month follow-up [134]. The effect was irrespective of the degree of left ventricular systolic impairment, and the performance of AF ablation resulted in greater odds of the improvement of left ventricular ejection fraction to over 35% [135]. Additionally, subjects with the less severe functional status exhibited the greatest improvement [135]. Another important aspect of this trial was the AF burden. Though similar at baseline and non-predictive of incident endpoints, the 6-month AF burden was significantly reduced in patients undergoing CA [136]. An AF burden of <50% was associated with a lower incidence of the primary endpoint (HR 0.33, 95% CI 0.15–0.71, *p* = 0.014) [136]. However, it appears that only a limited number of real-world HF patients with AF meet the trial inclusion criteria [137]. Despite that fact, patients not meeting the inclusion criteria may have a modest benefit from CA (HR 0.79, 95% CI 0.73–0.86, *p* < 0.001) [137]. On the other hand, patients meeting the exclusion criteria gain no benefit from CA (HR 0.97, 95% CI 0.81–1.17) [137].

CA was also evaluated in HF patients with a high burden of AF, characterized as either paroxysmal AF with more than four episodes in the previous 6 months, or persistent AF with a duration of less than 3 years. The performance of AF ablation was accompanied by an improvement in left ventricular systolic function and quality of life, as well as with a decrease in N-terminal pro-brain natriuretic peptide compared to rate control [138]. A trend towards a lower incidence of the primary endpoint was also noted with AF ablation (HR 0.71, 95% CI 0.49–1.03, *p* = 0.066) [138]. In another randomized clinical trial, CAMERA-MRI, CA in patients with persistent AF and systolic dysfunction led to ameliorated or even normalized left ventricular ejection fraction, especially in subjects without evidence of ventricular fibrosis [140]. The rate of AF recurrence and burden was 57% and 10.6%, respectively [140]. In the randomized AMICA trial, the improvements in left ventricular systolic function, natriuretic peptides, and quality of life were similar between CA and best medical therapy, despite a numerically lower AF burden [141]. In the less well-studied group of patients with HF and a preserved ejection fraction, CA may be equally effective at maintaining sinus rhythm compared to patients without HF, with a tendency to greater sinus rhythm maintenance and reduced HF hospitalizations compared to medical therapy [142,143].

## 3. Left Atrial Appendage Closure

Oral anticoagulation remains the cornerstone of stroke prevention in AF patients, with direct oral anticoagulants (apixaban, rivaroxaban, dabigatran, edoxaban) having replaced warfarin due to their superior safety and efficacy profile. However, for the subgroup of patients at very high risk for bleeding, alternative approaches need to be sought. LAAC is the most popular of these approaches, since the vast majority (>90%) of thrombi in nonvalvular AF originate in the LAA [144], which is a structure of variable shape and size with neurohormonal and reservoir functions. In AF, left atrial remodeling with alterations in shape, blood flow (stasis), and the presence of trabeculations are believed to be implicated in LAA thrombogenesis [145]. Surgical LAAC is a procedure with documented efficacy, as shown in the recently completed LAAOS-III randomized trial, as well as in a recent meta-analysis, for patients with AF undergoing cardiac surgery for another indication [146,147]. Percutaneous LAAC has also gained attention recently due to the safety and efficacy of the Watchman and Amplatzer devices (Table 2) [148]. Importantly, LAAC was noninferior to direct oral anticoagulants in terms of efficacy and safety in a randomized trial of high-risk patients [149]. Below, we review the current evidence in the field with the approved and under-investigation devices.

### 3.1. Percutaneous LAAC

#### 3.1.1. Watchman and Watchman FLX

After the initial shortcoming of the PLAATO device [150], the Watchman device was the first approved. The landmark trial that compared the Watchman device to warfarin in nonvalvular AF with CHADS_2_ score ≥1 showed a lower rate of the primary endpoint (stroke, systemic embolism, and cardiovascular/unexplained death) after a 3.8-year follow-up with the device implantation [151]. The device’s safety and efficacy have been further supported by the evaluation of the Continued Access to PROTECT-AF and Continued Access to PREVAIL registries [152]. A recently reported analysis of the National Cardiovascular Data Registry LAAO Registry highlighted acceptable 1-year rates of ischemic stroke, mortality, and major bleeding [153]. Watchman implantation also led to quality-of-life improvements compared to warfarin [154]. Moreover, the device implantation led to ameliorated left atrial function, evidenced by increased ejection fraction and peak atrial contraction strain [155].

Regarding potential complications, the presence of peridevice leak ≥5 mm should be considered, however, since it may be associated with an increased risk of thromboembolism [156,157]. Device-related thrombosis is also an infrequent complication that may be accompanied by a higher risk of thrombotic events [158]. Risk factors for device-related thrombosis have been identified, namely hypercoagulability disorders, pericardial effusion, renal impairment, implantation depth >10 mm from the pulmonary vein limbus, and nonparoxysmal AF [159]. Device embolization into the aorta, left atrial cavity, and left ventricle are other catastrophic complications that can occur intraoperatively or postoperatively [160]. Embolized devices can be rescued percutaneously or through surgical retrieval, especially in cases of device embolization in the left ventricle, mitral apparatus, and descending aorta [160].

A second-generation device, the Watchman FLX, aiming at a simpler implantation with full recapture and repositioning, was evaluated in 165 elderly patients with a mean CHA_2_DS_2_-VASc score of 4.4 [161]. Importantly, the majority of patients had a history of major bleeding, and one quarter of LAAs were considered to have complex morphology. The rate of procedure-related complications was low, and no periprocedural major adverse events were noted. Only 6.7% received anticoagulants at discharge. The rates of peridevice leak and device-related thrombosis were low in the short term. Similar results were reported from a single European center in 91 patients [162]. Ultimately, the PINNACLE FLX trial established its high efficacy and safety [163], even in populations with previously failed Watchman 2.5 implantation or prohibitive LAA anatomy [164]. Compared with the original Watchman device, Watchman FLX had a similar short- and mid-term safety profile, with a superior sealing rate [165,166,167].

#### 3.1.2. Amplatzer Cardiac Plug and Amulet

As early as 2003, the Amplatzer atrial septal occluder has been tried in patients with AF, demonstrating adequate efficacy and safety [168]. Early experiences with the Amplatzer cardiac plug (ACP) have been encouraging [169,170,171], and they were boosted by large registry data, showing high procedural success, low 1-year mortality and stroke/systemic thromboembolism rates, as well as few bleeding events [172]. At long-term follow-up, the ACP implantation was associated with lower ischemic stroke rates than those predicted by conventional risk scores [173]. Importantly, age and renal function should not be deterring factors, as the elderly and those with various degrees of renal dysfunction may exhibit similar efficacy and safety [174,175]. Adequate preoperative assessment with coronary-computed tomography angiography and follow-ups of patients with risk factors for device-related thrombosis (high ischemic risk scores, platelet count, and ejection fraction) need to be considered in these patients [176,177]. However, neither device-associated thrombosis nor peridevice leak were predictive of an increased risk of adverse cardiovascular events [178].

The next generation Amplatzer Amulet device, initially introduced in 2013, has a similar design to ACP, but offers easier implantation and reduced periprocedural complications. According to the results of a prospective global observational study including 1088 patients at high ischemic and bleeding risk who underwent its implantation, the rate of cardiovascular death or ischemic stroke was 8.7% at a 2-year follow-up [179]. This was accompanied by low rates of periprocedural major adverse events (4.0%), peridevice leak ≥3 mm (1.6%), and device-related thrombus (1.6%). Interestingly, a 67% reduction in the ischemic stroke rate compared to the predicted risk score was noted. Moreover, major bleeding event rates did not exceed 10%. The ischemic stroke risk reduction was more prominent in patients with CHA_2_DS_2_-VASc score ≥3 [180]. In addition, the results were similar across all age groups [181]. At 1-year follow-up, device-related thrombosis was encountered in 17 patients, and were associated with a greater risk of ischemic cerebrovascular events (HR 5.27, 95% CI 1.58017.55, *p* = 0.007), with a larger LAA orifice width being a predictive factor (HR 1.09, 95% CI 1.00–1.19, *p* = 0.04) [182].

When comparing the two Amplatzer LAAC devices, no major differences in the primary efficacy and safety endpoints were identified [183]. Interestingly, LAAC with Amplatzer devices led to a net clinical benefit compared with medical therapy in a propensity score-matched cohort study, by reducing the risk of stroke, systemic embolism, and cardiovascular/unexplained death (HR 0.70, 95% CI 0.53–0.95, *p* = 0.026) [184]. When the Amplatzer Amulet device was compared to direct oral anticoagulants, patients in the device group experienced fewer major bleeding events (HR 0.62, 95% CI 0.49–0.79), together with lower death rates (HR 0.53, 95% CI 0.43–0.64) [185]. Finally, although Amplatzer and Watchman devices appear to be similar in terms of efficacy and safety in previously reported registry data analyses [186,187], the lately published Amulet IDE randomized controlled trial pointed to a higher rate of periprocedural complications with the Amplatzer Amulet device compared to Watchman [188].

#### 3.1.3. Other Devices

Perhaps the most extensively studied device other than Watchman and Amplatzer is the LARIAT system, combining endocardial and epicardial manipulation, ultimately ligating the LAA. Although early studies suggested an efficient LAA closure, this was hindered by high rates of procedural complications such as pericardial effusion, pericarditis, and major bleeding [189,190]. Periprocedural colchicine administration may be able to mitigate pericardial damage, as shown in two studies [191,192]. Among the interesting observations made with the LARIAT system are the reduction in AF burden [193], and the reversal of adverse atrial remodeling even in cases of incomplete closure [194,195,196]. Regarding major clinical endpoints, LARIAT was effective in reducing thromboembolic and bleeding events, as well as mortality rate, compared to a control group [197,198,199]. However, small leaks, which are not uncommon, should be considered, as they are predictive of adverse outcomes [200,201]. Sealing leaks with occluders may be a reasonable option in this scenario [202]. Compared to endocardial LAAC devices, the LARIAT system is associated with a higher short-term complication rate [203], but similar mid-term efficacy [204]. The second-generation LARIAT^+^ has improved features, such as snare expansion from 40 mm to 45 mm, the addition of a platinum–iridium ‘L’ Marker that allows identification of the device orientation under fluoroscopy, and a stainless steel wire braid on catheter shaft that provides improved ‘torque-ability’ of the catheter. Initial reports from registries highlight the high rates of acute and short-term LAAC, paired with low periprocedural complications and few thromboembolic events at longer term follow-up [205,206].

The LAmbre is a relatively new device that may be helpful in occluding large LAA, with its additional stabilization mechanism which catches the LAA trabeculations using its eight claws. The implantation success rate was 89.9% [207], with peridevice leak being detected in 17% at 1-year follow-up in one study [208]. This was not accompanied by a higher thromboembolic risk, however [208], perhaps due to the absence of significant leaks. Device-related thrombosis was rare, and no device embolization was documented [207]. Concerning mid-term outcomes, there was a low risk of death or ischemic cerebrovascular events, and major bleeding was seen in 11% of the patients at 1-year follow-up [209]. In a study with a mean follow-up of 37.8 months, the rates of death, stroke, and device-related thrombus were 7.1%, 3.6%, and 3.6%, respectively [210]. When compared to the Amplatzer and Watchman devices, the clinical efficacy and safety endpoints were met with similar frequency [211,212].

## 4. Conclusions

The milestones achieved in percutaneous approaches for atrial fibrillation, namely catheter ablation and left atrial appendage closure, constitute safe and effective treatment modalities in rhythm control and stroke prevention, respectively. Although currently reserved for a carefully selected group of patients, continuous research and persistently positive clinical trial data in a broad range of atrial fibrillation patients may mandate the need for the expansion of their indications.

## Figures and Tables

**Table 1:** Landmark randomized clinical trials in patients with atrial fibrillation (AF) and heart failure (HF).

Trial	Patient Number	Study Population	Control Group	Follow-up	Primary Endpoint	Other
CASTLE-AF [134,135,136]	363	Symptomatic AF HF (LVEF < 35%) NYHA class II-IV ICD	AADs	37.8 months	All-cause mortality or HF hospitalization (HR 0.62, 95% CI 0.43–0.87, *p* = 0.007)	Effect irrespective of LVEF Less severe functional status led to greater improvement ↓ AF burden
RAFT-AF [138]	411	High-burden PAF or PeAF NYHA class II-III Elevated NT-proBNP	MRC	37.4 months	All-cause mortality or HF events (HR 0.71, 95% CI 0.49–1.03, *p* = 0.066)	↑ LVEF ↓ NT-proBNP ↑ QoL ↑ 6MWT
CAMERA-MRI [139,140]	68	Persistent AF LVEF ≤ 45% without identifiable cause	MRC	6 months	Change in cMRI-LVEF (MD 14.0, 95% CI 8.5–19.5, *p* < 0.0001) LVEF normalization (CA 58% vs. MRC 9%, *p* = 0.0002)	↓ NYHA class ↓ BNP ↓ LAVi ↓ AF recurrence-burden
AMICA	140	PeAF LVEF ≤ 35%	BMT	12 months	Increase in LVEF (No difference)	↓ AF recurrence-burden ↔ BNP ↔ 6MWT ↔ QoL

LVEF: left ventricular ejection fraction, NYHA: New York Heart Association, ICD: implantable cardioverter defibrillator, HR: hazard ratio, CI: confidence interval, PAF: paroxysmal AF, PeAF: persistent AF, NT-proBNP: N-terminal pro-hormone brain natriuretic peptide, MRC: medical rate control, QoL: quality of life, 6MWT: 6-min walk test, cMRI: cardiac magnetic resonance imaging, MD: mean difference, CA: catheter ablation, LAVi: left atrial volume index, BMT: best medical therapy. ↓ indicates a decrease, ↑ indicates an increase, ↔ indicates no change

**Table 2:** Characteristics and clinical outcomes of the most well-studied left atrial appendage (LAA) closure devices.

	ACP	Amulet	WM	WM FLX
Description	Self-expanding, double-disc device consisting of a nitinol mesh with polyester fabric		Self-expanding nitinol 10-strut frame with a 160-μm polyethylene terephthalate fabric mesh cap	
Device size, mm	16-30	16-34	21–33	20–35
Implant success (%)	97.3	99.1	95.1	98.8
Periprocedural MAE (%)	5.0	4	2.2	0.5
Device embolization (%)	0.8	0.2	0.7	0
DRT (%)	4.4	1.6 (1-year)	3.7	1.8
Risk factor for DRT		Large LAA orifice width	Hypercoagulability disorders Pericardial effusion Renal impairment Implantation depth >10 mm from the pulmonary vein limbus Nonparoxysmal AF	NA
PDL ≥ 3 mm (%)	1.9	1.6	13.1	7.4 (>0mm)
All-cause mortality (%)	4.2 (1-year)	NA	3.6 (5-year)	6.6 (1-year)
Ischemic stroke (%)	0.9 (1-year)	2.2/year	1.6 (5-year)	2.6 (1-year)
Major bleeding (%)	1.5 (1-year)	7.2/year	1.7 (5-year)	7.9 (1-year)

ACP: Amplatzer cardiac plug, WM: Watchman, MAE: major adverse events, DRT: device-related thrombosis, PDL: peridevice leak, AF: atrial fibrillation, NA: not available.

## Data Availability

Not applicable.
